# Porous Aluminum Oxide and Magnesium Oxide Films Using Organic Hydrogels as Structure Matrices

**DOI:** 10.3390/nano8040186

**Published:** 2018-03-22

**Authors:** Zimei Chen, Dirk Kuckling, Michael Tiemann

**Affiliations:** 1 Department of Chemistry—Organic and Macromolecular Chemistry, Paderborn University, 33098 Paderborn, Germany; zimei.chen@uni-paderborn.de; 2 Department of Chemistry—Inorganic Functional Materials, Paderborn University, 33098 Paderborn, Germany

**Keywords:** mesoporous, Al_2_O_3_, MgO, poly(dimethylacrylamide), hydrogel, thin film, spin coating, SEM, FIB, Kr physisorption

## Abstract

We describe the synthesis of mesoporous Al_2_O_3_ and MgO layers on silicon wafer substrates by using poly(dimethylacrylamide) hydrogels as porogenic matrices. Hydrogel films are prepared by spreading the polymer through spin-coating, followed by photo-cross-linking and anchoring to the substrate surface. The metal oxides are obtained by swelling the hydrogels in the respective metal nitrate solutions and subsequent thermal conversion. Combustion of the hydrogel results in mesoporous metal oxide layers with thicknesses in the μm range and high specific surface areas up to 558 m^2^∙g^−1^. Materials are characterized by SEM, FIB ablation, EDX, and Kr physisorption porosimetry.

## 1. Introduction

The synthesis of metal oxides with uniform mesopores is often achieved by utilization of porogenic structure directors or matrices. For example, micellar aggregates of amphiphilic species—such as surfactants or block co-polymers—are frequently utilized as porogens. They form spontaneously by self-organization and serve as pore fillers or even as structure-directing species during the formation of the inorganic phase by a sol–gel-based synthesis (‘soft templating’) [1,2]. This synthesis method is applicable to a limited variety of inorganic products, such as silica and some other oxidic materials, including aluminum oxide (Al_2_O_3_) [3,4,5]. For uniform, continuous layers (‘solid films’) of mesoporous metal oxides at a substrate surface the soft-templating approach is usually the method of choice, because the spontaneous self-aggregation into micellar units can take place inside a liquid film that contains both the amphiphilic species and the inorganic precursor compounds. For this purpose, the micellization is induced by evaporation of the solvent (evaporation-induced self-assembly, EISA) [6,7]. It needs to be stressed, though, that several metal oxides cannot be obtained in this way as their formation may go along with phase-separation and segregation from the amphiphilic species. As an alternative, the concept of using solid, porous structure matrices (‘hard templates’) has been shown to be a more versatile option [8,9]. This method, often called ‘nanocasting’, comprises the synthesis of the desired products within the pores of a silica or carbon matrix, followed by selective removal of the matrix; the product is obtained as a ‘replica’ of the pore system in the matrix. Nanocasting can be used for the fabrication of a multitude of metal oxides, including Al_2_O_3_ [10,11,12], as well as those that have so far not been obtained by soft templating, e.g., magnesium oxide (MgO) [12,13,14,15]. However, the nanocasting concept still has its limitations when it comes to the synthesis of porous films, since the removal of the structure matrix may cause detachment of the replica film from the substrate.

We have recently described the synthesis of mesoporous metal oxides by using poly(dimethylacrylamide) hydrogels as matrices [16,17]. Hydrogels are three-dimensional structures composed of hydrophilic polymer chains, which can absorb and hold large quantities of water in the spaces between the chains [18]. They can be fabricated via physical or chemical cross-linking [19] and have been used as matrices for porous inorganic materials [16,17,20,21,22]. Their utilization as porogenic matrices may be regarded as halfway between ‘soft’ and ‘hard templating’. The hydrogel forms a continuous network that can take up the inorganic precursor species (such as a metal salt) with no risk of phase-separation, similar to a hard structure matrix. At the same time, the swollen hydrogel is a highly flexible phase; the (cross-linked) polymer strands are more or less loosely arranged and displaceable, like a soft matrix. In fact, the porogenic impact may even occur when the water-soluble polymer strands are not even cross-linked, but only sterically entangled [23]. We have rationalized that thick bundles of polymer chains (rather than single, individual chains) in poly(dimethylacrylamide) hydrogels form the porogenic entities [16]. The products obtained so far were powders with somewhat uniform mesopores and high specific surface areas.

**Scheme 1:**
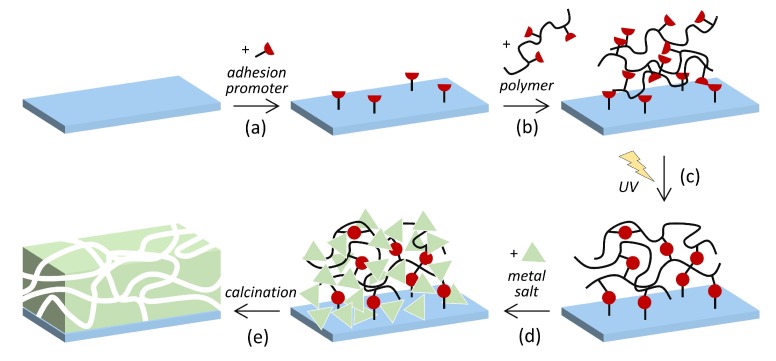
Preparation of porous metal oxide (Al_2_O_3_, MgO) layers: (**a**) anchoring of the adhesion promoter on the Si wafer substrate; (**b**) spreading of the polymer by spin coating; (**c**) hydrogel formation and immobilization on the substrate by photo-induced cross-linking; (**d**) swelling in metal salt solution (Al(NO_3_)_3_, Mg(NO_3_)_2_); (**e**) formation of the porous metal oxide and combustion of the hydrogel by calcination.

Here we report on utilizing the same kind of porogenic hydrogels for mesoporous layers (solid films) of aluminum oxide (Al_2_O_3_) and magnesium oxide (MgO) at the surface of silicon wafer substrates. Photo-cross-linked poly(dimethylacrylamide) hydrogels are attached to the substrate by chemical bonding and serve as matrices for the metal oxides (Scheme 1). Porous Al_2_O_3_ and MgO with a high surface-to-volume ratio play an important role in separation [24,25] and heterogeneous catalysis [26,27,28,29]. Especially for the latter application immobilized layers of the catalyst (MgO) or support (Al_2_O_3_) materials with large pores are considered advantageous to facilitate easy access of the reactants by diffusion.

## 2. Materials and Methods

Materials: Acryloyl chloride (Alfa Aesar, Karlsruhe, Germany, 96%), allylamine (Sigma-Aldrich, Taufkirchen, Germany, 98%), aluminum nitrate nonahydrate (Sigma-Aldrich, ≥98.0%), ammonia solution (Stockmeier, Bielefeld, Germany, 25%), bicyclohexyl (Acros, Geel, Belgium, 99%), chloroform (Stockmeier), chlorodimethylsilane (Alfa Aesar, 97%), 1,2-diaminoethane (Acros, >99%), 2,3-dimethylmaleic anhydride (Acros, 97%), di-*tert*-butyl dicarbonate (Boc_2_O, Acros, 97%), ethanol, absolute (Sigma-Aldrich), hydrochloric acid, conc. (Stockmeier, 37%), hydrogen peroxide (Stockmeier, 35%), magnesium nitrate hexahydrate (Sigma-Aldrich, ≥97%), magnesium sulfate (Grüssing, Filsum, Germany, 99%), platinum(0)-1,3-divinyl-1,1,3,3-tetramethyldisiloxane complex solution in xylene (Sigma-Aldrich, Pt ~2%), 4″-silicon-wafer (Plano, Wetzlar, Germany), sulfuric acid, conc. (Stockmeier, ≥98%), thioxanthone (Sigma-Aldrich, 98%), and triethylamine (TEA, Grüssing, 99%) were used as received. Acetone (Stockmeier), diethyl ether (Hanke+Seidel, Steinfurt, Germany), ethyl acetate (Stockmeier), *n*-hexane (Stockmeier), methanol (Stockmeier), *n*-pentane (Stockmeier), silica gel (VWR), sodium bicarbonate (Stockmeier), and sodium chloride (Stockmeier) were of technical grade and used as received. 1,4-Dioxane (Carl Roth, Karlsruhe Germany, ≥99.5%), *N*,*N*-Dimethylacrylamide (DMAAm, TCI, Eschborn, Germany, 99%), tetrahydrofuran (THF, BASF, Ludwigshafen, Germany), and toluene (Grüssing, 99.5%) were distilled under low pressure. α,α′-Azobisisobutyronitrile (AIBN, Fluka, Seelze, Germany, >98%) was recrystallized from methanol. Cyclohexanone (Sigma-Aldrich, ≥99.0%) was distilled. Dichloromethane (Stockmeier) was dried over CaCl_2_ and distilled. 

Characterization: ^1^H and ^13^C NMR spectra were recorded on a Bruker AV 500 spectrometer at 500 MHz and 125 MHz, respectively. Reference solvent signals at 7.26 and 2.56 ppm were used for spectra in CDCl_3_ (99.8 atom % Deuterium) and DMSO-d_6_ (O=S(CD_3_)_2_, 99.9%), respectively. Gel permeation chromatography (GPC) was performed in chloroform for PDMAAm at 30 °C and at a flow rate of 0.75 mL∙min^−1^ on a Jasco 880-PU Liquid Chromatograph connected to a Shodex RI-101 Detector. The instrument was equipped with four consecutive columns (PSS-SDV columns filled with 5 μm gel particles with a defined porosity of 10^6^ Å, 10^5^ Å, 10^3^ Å and 10^2^ Å, respectively) and were calibrated by poly(methyl methacrylate) standards. Krypton (Kr) physisorption analysis was performed at 77 K on a Quantachrome Autosorb 6B instrument. The masses of the films were determined by weighing the wafer substrates before and after film synthesis. Several samples of identical films (7 × 7 mm substrate dimensions) were combined for each sorption measurement to provide sufficient overall film masses (1–200 mg). Samples were degassed at 120 °C for 12 h prior to measurement. The specific surface areas were assessed by multi-point BET analysis [30] in the range 0.1 ≤ *p/p*_0_ ≤ 0.3. Scanning electron microscopy (SEM) and energy-dispersive X-ray (EDX) spectroscopy were performed on a Zeiss NEON^®^ 40 microscope connected with an UltraDry detector from Thermo Fisher Scientific (Waltham, MA, USA).

Cross-Linker Synthesis: 2-(Dimethyl maleimido)-*N*-ethyl-acrylamide (DMIAAm) was synthesized through a four-step reaction as described in the literature [31] and can be found in detail in the Supplementary Materials.

Polymer Synthesis: Poly(DMAAm-*co*-DMIAAm) was synthesized with DMAAm monomer and DMIAAm cross-linker by free radical polymerization initiated with AIBN in an analogous fashion as described in the literature [31]. DMAAm (95 mol %) and DMIAAm (5 mol %) and about 0.002 mol % AIBN relative to the total amount of monomer were dissolved in 1,4-dioxane and purged with argon for 20 min. The total monomer concentration was 1 mol∙L^−1^. The polymerization was carried out at 70 °C for 7 h under argon atmosphere. Afterwards, the polymer was precipitated in diethyl ether and re-precipitated from tetrahydrofuran into diethyl ether for purification. Finally, the polymer was dried in high vacuum and characterized by NMR spectroscopy and GPC. ^1^H NMR (500 MHz, CDCl_3_): δ (ppm) = 1.51–1.83 (m, CH_2_), 1, 94 (s, CH_3_), 2.3–2.75 (m, CH), 2.77–3.19 (m, N-CH_2_, NH-CH_2_, N-CH_3_), 3.6 (b, NH). Yield: 88%, *M_n_*: 39,000 g∙mol^−1^, DMIAAm composition: 5 mol % (Feed)/4.8 mol % (NMR), PD: 5.1.

Synthesis and Immobilization of the Adhesion Promoter: 1-[3-(Chloro-dimethyl-silanyl)-propyl]-3,4-dimethyl-maleimide was synthesized as described in the literature [32] (see Supplementary Materials). A Si wafer (7 mm × 7 mm) was activated with a mixture (7:3 vol.) of concentrated sulfuric acid (H_2_SO_4_) and 30% hydrogen peroxide (H_2_O_2_) solution at 90 °C for 1 h. After repeated rinsing with water and ethanol and drying in argon stream the adhesion promoter was absorbed from 1 vol % solution in bicyclohexyl for 24 h. Finally the wafer was rinsed with chloroform and abs. ethanol and dried in an argon flow.

Preparation of Hydrogel Films: Solutions of the polymer in cyclohexanone (2 mL) with 2 wt % thioxanthone as a sensitizer were spin-coated on a pre-treated Si wafer, using variable spin velocities and polymer concentrations (see Results and Discussion section); polymer solutions were first spread at 250 rpm for 25 s, followed by 60 s of spinning at the final velocity. The polymer layer on the wafer was irradiated with UV light for one minute by using a 200 W mercury short arc lamp with an intensity of 266 mW∙cm^−2^.

Preparation of Porous Al_2_O_3_ and MgO layers: For the preparation of Al_2_O_3_, the hydrogel film was re-swelled in saturated aqueous aluminum nitrate solution (1.9 mol∙L^−1^) overnight and then treated with the vapor of an aqueous ammonia solution (12.5 wt %) for 3 h at 60 °C to convert Al(NO_3_)_3_ to Al(OH)_3_/AlO(OH), followed by drying overnight at 60 °C. The material was calcined in a tube furnace for 4 h at 500 °C with a heating rate of 1 °C∙min^−1^ to combust the polymer and to form a porous Al_2_O_3_ film on the Si wafer. For the preparation of MgO the hydrogel film was re-swelled in saturated aqueous magnesium nitrate solution (4.9 mol∙L^−1^) overnight and then dried at 120 °C. The material was calcined in a tube furnace for 2 h at 300 °C and 2 h at 500 °C with a heating rate of 1 °C∙min^−1^ to combust the polymer and to form a porous MgO film on the Si wafer.

In an alternative approach, the above-described preparation of the hydrogel film was modified by dissolving the polymer in methanol (instead of cyclohexanone) and by adding aluminum nitrate or magnesium nitrate to this solution before (instead of after) spin-coating (2500 rpm final spin velocity) and subsequent photo-cross-linking. Otherwise, the same synthesis protocol was used.

## 3. Results and Discussion

**Scheme 2:**
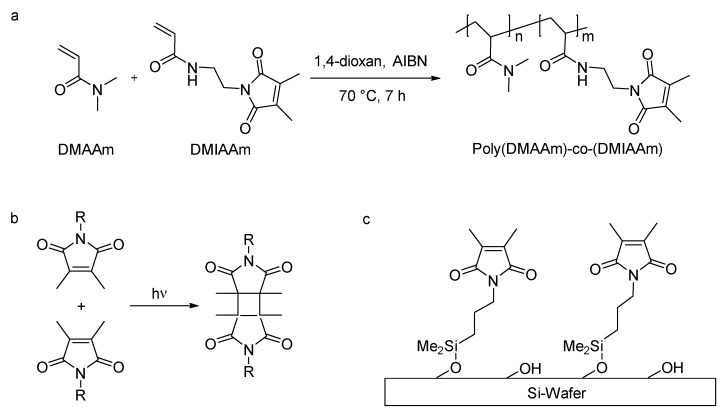
(**a**) Synthesis of the polymer serving as the precursor for the hydrogel films; (**b**) photo-cross-linking of the polymer; (**c**) schematic [19] of the adhesion promoter attachment to the Si wafer surface.

Photo-cross-linked hydrogel films were used as structure matrices for the preparation of porous alumina (Al_2_O_3_) and magnesia (MgO) layers. The polymer for the hydrogels was synthesized by free radical polymerization of *N*,*N*-dimethylacrylamide (DMAAm) and 2-(dimethyl maleimido)-*N*-ethyl-acrylamide (DMIAAm) (Scheme 2a). The synthesized polymers have a molecular weight (*M_n_*) of ca. 39,000 g∙mol^−1^. DMIAAm served as a photo-cross-linker to form a three-dimensional polymer network (Scheme 2b) by a reaction mechanism that can be primarily described as a [2+2] cycloaddition; however, other mechanisms are also possible [32]. According to NMR data the DMIAA fraction in the polymers is 4.8 mol %, slightly less than the feed composition (5 mol %), which is in accordance with previous findings [33]. To covalently attach the hydrogel to a silicon wafer substrate, 1-[3-(chloro-dimethyl-silanyl)-propyl]-3,4-dimethyl-maleimide was used as an adhesion promoter. The promoter was applied to the wafer prior to coating with the polymer. For this purpose, the wafer surface was chemically activated by oxidative treatment with piranha solution (H_2_SO_4_/H_2_O_2_). The promoter bonds to the surface via its reactive chloro-silane function (Scheme 2c); the maleimide function can react with the polymer during photo-induced cross-linking.

**Figure 1:**
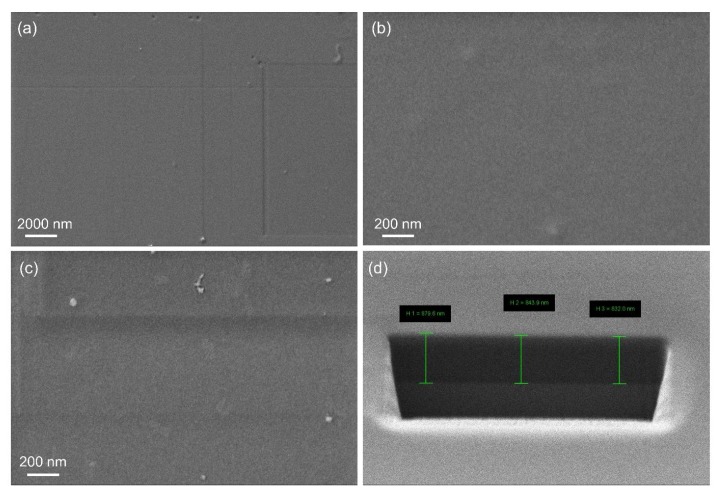
SEM images of dry hydrogel films prepared by spin-coating with variable polymer concentration and spin velocity (average film thicknesses: (**a**) 0.187 μm; (**b**) 0.588 μm; (**c**,**d**) 0.851 μm; see Table 1). Image (**d**) shows an example of the FIB ablation analysis of a film (average thickness: 0.851 μm; green bars: 0.8796, 0.8439, 0.8320 μm).

Porous Al_2_O_3_ or MgO layers were created by pre-fabricating hydrogel films on the substrate and then adding the inorganic precursor species in a second step (Scheme 1). The polymer was spin-coated on the pretreated Si wafer by using cyclohexanone as a solvent. The polymer concentration and spin velocity were varied in order to obtain variable film thicknesses. Photo-cross-linking of the polymer film was then achieved by UV irradiation as described in the Experimental Section. The cross-linked network forms a thin hydrogel film at the Si wafer surface. Figure 1 shows example scanning electron microscopic (SEM) images of dry films exhibiting high degrees of homogeneity. (further examples are shown in Figure S1 in the Supplementary Materials). The film thicknesses were analyzed by focused ion beam (FIB) ablation. Figure 1d shows a rectangular hole cut out of the film. The image was taken from a tilted angle (ca. 45° to the film surface), showing both the section through the film and the underlying substrate. This way, the average thickness of the film can be measured; depending on polymer concentration and spin velocity it ranges from 0.187 μm to 0.851 μm (Table 1).

The hydrogel film was then impregnated with Al(NO_3_)_3_ or Mg(NO_3_)_2_ by swelling in a saturated aqueous solution of the respective salt. The Al salt was transformed to Al(OH)_3_/AlO(OH) by exposure to ammonia vapor and subsequently calcined to create Al_2_O_3_; this procedure is frequently applied for the structure-directed synthesis of Al_2_O_3_ [10,11]. The Mg salt was directly transformed to MgO by calcination. In both cases, the calcination procedure leads to the thermal combustion of the hydrogel matrix, leaving behind metal oxide layers that remain attached to the Si wafer substrates (presumably by Si-O-Al bonds in case of Al_2_O_3_ and by ionic interaction with the charged oxidized Si surface in case of MgO, respectively). Identification of the metal oxide phases by XRD was not feasible due to the very low thickness of the layers (see below), but previous studies [16,17,23] have shown that the applied synthesis conditions lead to formation of γ-Al_2_O_3_ (with low crystallinity) and MgO, respectively.

**Figure 2:**
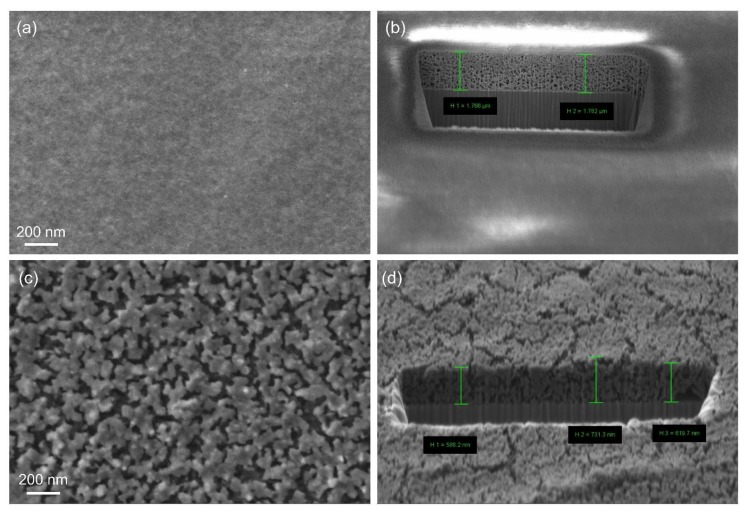
SEM images and FIB ablation analysis of two example layers of Al_2_O_3_ ((**a**,**b**); average thickness: 1.77 μm, prepared with a hydrogel film of 0.588 μm thickness; green bars: 1.766 and 1.782 μm) and MgO ((**c**,**d**); average thickness: 0.646 μm, prepared with a hydrogel film of 0.851 μm thickness; green bars: 0.5862, 0.7313, 0.6197 μm).

Figure 2 shows SEM images with FIB analysis of two examples of porous Al_2_O_3_ and MgO layers. (further examples are shown in Figure S2 in the Supplementary Materials) EXD analysis confirms the approximate stoichiometry of Al/O = 1.5 and Mg/O = 1, respectively (Table S1 in the Supplementary Materials). The Al_2_O_3_ layer (Figure 2a,b) exhibits a fairly smooth and homogeneous texture and an average thickness of 1.77 μm, three times the thickness of the non-swollen (dry) hydrogel film that was used as the matrix (0.588 μm). This difference reflects the swelling of the hydrogel and also indicates a certain degree of porosity in the Al_2_O_3_ layer, as will be substantiated below.

**Figure 3:**
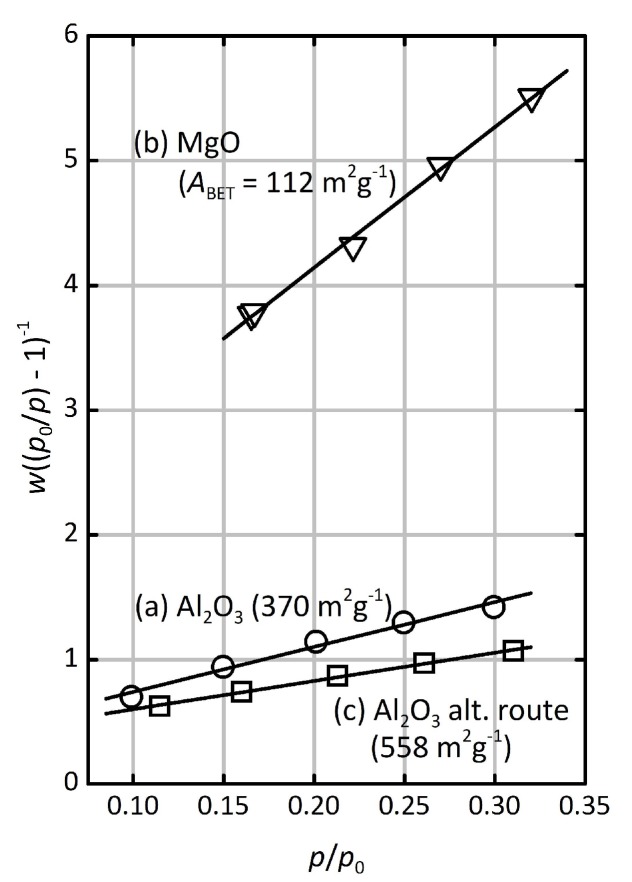
BET plots of Kr physisorption data of porous metal oxide layers: (**a**) Al_2_O_3_ layer (1.77 μm thickness); (**b**) MgO (0.646 μm); (**c**) Al_2_O_3_ (prepared by pre-mixing the polymer).

Assessment of the pore size distribution by nitrogen (N_2_) or argon (Ar) physisorption analysis was not possible due to the low overall amount of material (as frequently encountered for thin layers of porous material), but krypton (Kr) physisorption allowed a five-point BET analysis, as shown in Figure 3a. The isotherm showing the adsorbed amount of Kr is shown in Figure S3 in the Supplementary Materials. The specific surface area of the Al_2_O_3_ layer is 370 m^2^∙g^−1^, corresponding to 0.259 m^2^∙cm^−2^ if normalized to the covered area of the substrate. The latter value incorporates the respective film thickness, while the former value is independent of the film dimensions. This large surface area confirms that the Al_2_O_3_ layer is indeed porous. As mentioned in the Introduction section, we have recently reported on the synthesis of γ-Al_2_O_3_ materials synthesized by the same procedure (using the same type of hydrogels), but in form of powders rather than as thin layers [16,17,23]. The powder samples exhibited similar BET surface areas (250–370 m^2^∙g^−1^) with narrow pore size distributions around ca. 4 nm and mesopore volumes in the range of 0.4–0.5 cm^3^∙g^−1^. Hence, it is fair to assume similar mesopores for the Al_2_O_3_ layer presented here. The origin of these mesopores is the porogenic impact of bundles of polymer strands in the hydrogel; the combustion of the hydrogel creates disordered, tubular mesopores, as previously described [16]. The MgO layer (Figure 2c,d), on the other hand, is significantly less homogeneous than the Al_2_O_3_ layer; it exhibits a rough surface with raptures and an almost granular texture. Its average thickness is 0.646 μm, which is actually less than the thickness if the respective non-swollen (dry) hydrogel film (0.851 μm). This indicates a lower degree of porosity which is confirmed by a low BET surface area of 112 m^2^∙g^−1^ (0.025 m^2^∙cm^−2^; Figure 3b). Obviously, the polymer network does not have a strong porogenic impact in this case. This may be due to the fact that the hydrogel matrix starts to decompose before a sufficiently stable network of MgO has formed.

**Figure 4:**
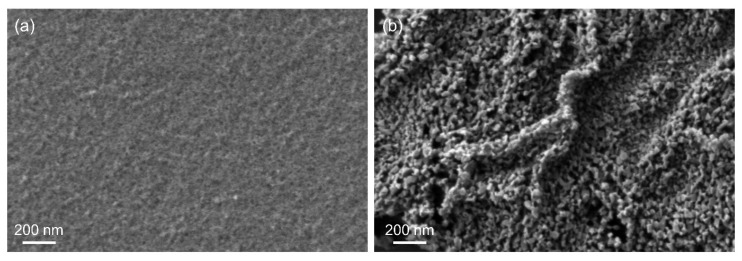
SEM images of two example layers of (**a**) Al_2_O_3_ and (**b**) MgO prepared by pre-mixing the polymer ((a): 200 mg; (b): 150 mg) with the metal salts ((a): 600 mg Al(NO_3_)_3_·9H_2_O; (b): 450 mg Mg(NO_3_)_2_·6H_2_O) prior to spin-coating and subsequent photo-cross-linking and calcination.

In an alternative synthesis approach, we simplified the process by dissolving the metal salts and the precursor polymer in methanol before applying them to the silicon wafer by spin-coating and subsequent photo-cross-linking of the polymer. Hence, no drying of the hydrogel films and subsequent re-swelling in metal salt solutions is necessary which facilitates the overall procedure. The rest of the synthesis was carried out in the same way as before. Two example SEM images of the resulting Al_2_O_3_ and MgO layers are shown in Figure 4. Further examples are listed in Table S2 and shown in Figure S4 in the Supplementary Materials. The Al_2_O_3_ layer (Figure 4a) exhibits an average thickness of 0.364 μm and shows a fairly homogeneous texture, although not as smooth as in case of the synthesis procedure described above (i.e., by re-swelling the pre-fabricated hydrogel films with metal salt solutions). However, it exhibits a higher BET surface area of 558 m^2^∙g^−1^. Figure 3c; the surface area per substrate area, 0.080 m^2^∙cm^−2^, is lower as a consequence of a lower layer thickness. The MgO layer (Figure 4b) is even less homogeneous; it appears seriously granular and rough, with a similar BET surface area as for the material prepared by the first route (112 m^2^∙g^−1^). In summary, the alternative synthesis approach, despite being simpler and easier to carry out, cannot be regarded as equally successful as the first route in terms of the homogeneity and smoothness of the resulting metal oxide layers.

## 4. Conclusions

In summary, we have shown that the concept of using poly(dimethylacrylamide) hydrogels as porogenic matrices for the synthesis of mesoporous metal oxides can be applied to the preparation of porous layers on silicon substrates by anchoring the hydrogel to the substrate via chemical bonding. Homogeneous mesoporous layers of Al_2_O_3_ with high specific surface areas (up to 558 m^2^∙g^−1^) are obtained. The MgO layers display lower homogeneity and porosity.

## Figures and Tables

**Table 1:** Characteristics of dry hydrogel films obtained by spin-coating of the polymer and subsequent photo-cross-linking.

Spin Velocity (rpm)	Polymer Conc. (wt %)	Film Thickness (μm)
2500	5	0.187
2500	7.5	0.306
2500	10	0.588
1000	5	0.607
1000	7.5	0.801
1000	10	0.851

## Supplementary Materials

The following are available online at http://www.mdpi.com/2079-4991/8/4/186/s1, Figure S1: SEM images of dry hydrogel films, Figure S2: SEM images of Al_2_O_3_ and MgO layers, Figure S3: Kr physisorption isotherms, Figure S4: SEM images of Al_2_O_3_ and MgO layers prepared by pre-mixing, Table S1: EDX analysis, Table S2: Amounts of polymer and metal salts for pre-mixing synthesis.
