# Supplementary material for: Porous Aluminum Oxide and Magnesium Oxide Films Using Organic Hydrogels as Structure Matrices
Source: Nanomaterials (Basel). 2018 Mar 22;8(4):186. doi: 10.3390/nano8040186 (PMC5923516; doi:10.3390/nano8040186)
Supplement: Supplementary file 1 [file nanomaterials-08-00186-s001.pdf]

Porous Aluminum Oxide and Magnesium Oxide Films by Using Organic Hydrogels as Structure Matrices

Zimei Chen, Dirk Kuckling* and Michael Tiemann*

Supporting Information

Cross-linker Synthesis:

Step 1: <u>*tert*-Butyl-*N*-(2-aminoethyl)-carbonate</u>
A solution of Di-*tert*-butyldicarbonat (16.69 g, 0.077 mol) in 140 mL 1,4-dioxane was added dropwise to a solution of 1,2-diaminoethane (35.0 mL, 0.52 mol) in 130 mL 1,4-dioxane over a time span of 3 h. The mixture was stirred for 2 d at room temperature, the resulting precipitate was filtrated and the solvent was evaporated under reduced pressure. 260 mL water was added to the crude product and the precipitate was filtrated again. After that, the aqueous solution was saturated with sodium chloride and extracted eight times with 130 mL dichloromethane. The organic phase was dried with magnesium sulfate and the solvent removed under reduced pressure to yield 8.91 g of a colorless liquid. Yield: 73%. ^{1}H NMR (500 MHz, $CDCl_3$): δ (ppm) = 1.30 (s, 2H, NH_2), 1.40 (s, 9H, $(CH_3)_3$), 2.75 (t, ^{3}J = 5.98 Hz, 2H, NH_2-CH_2), 3.16 (q, ^{3}J = 5,78 Hz, 2H, NH-CH_2), 5.00 (br, 1H, NH). ^{13}C NMR (125 MHz, $CDCl_3$): δ (ppm)= 28,4 (CH_3), 41,9 (NH_2-CH_2), 43,4 (NH-CH_2), 79,1 (C), 156,2 (C=O).

Step 2: <u>*N*-[2-(3,4-Dimethyl-2,5-dioxo-2,5-dihydro-pyrrol-1-yl)-aminoethyl]-*tert*-butyl-carbonate</u>
To a solution of 2,3-dimethylmaleic anhydride (7.0 g, 0.056 mol) in 110 mL toluene a solution of *tert*-Butyl-*N*-(2-aminoethyl)-carbonate (8.91 g, 0.056 mol) in 37 mL toluene was added dropwise. The mixture was heated at 130 °C for 3 h using a reflux condenser equipped with a water trap. The solvent was removed under reduced pressure to obtain a yellow oil. The oil was dissolved in 40 mL toluene and dropped into 600 mL *n*-pentane. The precipitate was washed with 300 mL *n*-pentane and dried in a vacuum to obtain 9.51 g of white solid. Yield: 64%. ^{1}H NMR (500 MHz, $CDCl_3$): δ (ppm) = 1.39 (s, 9H, $(CH_3)_3$), 1.95 (s, 6H, CH_3), 3.29 (m, 2H, NH-CH_2), 3.60 (t, ^{3}J = 5,60 Hz, 2H, N-CH_2), 4.81 (s, 1H, NH). ^{13}C NMR (125 MHz, $CDCl_3$): δ (ppm)= 8,7 (CH_3), 28,3 ($(CH_3)_3$), 38,0 (N-CH_2), 39,8 (NH-CH_2), 79,3 (C), 137,3 (C=C), 155,9 (HN-C=O), 172,2 (N-C=O).

Step 3: <u>*N*-[2-(3,4-Dimethyl-2,5-dioxo-2,5-dihydro-pyrrol-1-yl)]-ethylamine hydrochlorid</u>
A suspension of *N*-[2-(3,4-Dimethyl-2,5-dioxo-2,5-dihydro-pyrrol-1-yl)-aminoethyl]-*tert*-butyl-carbonate (9.51 g, 0.036 mol), 92 mL ethyl acetate and 7 ml conc. hydrochloric acid was stirred for 20 h at room temperature. The precipitate was collected, washed five times with 30 mL ethyl acetate and dried in a vacuum to yield 6.71 g of a white solid. Yield: 92%. ^{1}H NMR (500 MHz, DMSO-d_6): δ (ppm) = 1.90 (s, 6H, 2 CH_3), 2.97 (t, ^{3}J = 6.15 Hz, 2H, CH_2-NH_3Cl), 3.65 (t, ^{3}J = 6.15 Hz, 2H, N-CH_2), 7.98 (br, 3H, NH_3Cl). ^{13}C NMR (125 MHz, $CDCl_3$): δ (ppm)= 8,9 (CH_3), 35,6 (NH_3Cl-CH_2), 38,0 (N-CH_2), 137,4 (C=C), 172,1 (C=O).

Step 4: <u>*N*-[2-(3,4-Dimethyl-2,5-dioxo-2,5-dihydro-pyrrol-1-yl)-ethyl]-acrylamide (DMIAAm)</u>
A mixture of *N*-[2-(3,4-Dimethyl-2,5-dioxo-2,5-dihydro-pyrrol-1-yl)]-ethylamine hydrochloride (6.71 g, 0.033 mol), triethylamine (18.4 mL, 0.131 mol) and acryloyl chloride (6.6 mL, 0.082 mol) in 330 mL tetrahydrofuran was stirred 2 h under argon atmosphere at room temperature. The precipitate was removed and the solvent was evaporated under reduced pressure. The remaining solid was dissolved in chloroform and washed three times with 160 mL water, two times with 160 mL saturated sodium bicarbonate and three times with 160 mL 0.1 M hydrochloric acid. The organic layer was dried over $MgSO_4$ and the solvent evaporated under reduced pressure. The product was purified by column chromatography with silica gel and ethyl acetate/*n*-hexane (2:1) to yield 4.41 g of a white solid. Yield: 60%. ^{1}H NMR (500 MHz, $CDCl_3$): δ (ppm) = 1.97 (s, 6H, 2 CH_3), 3.52 (m, 2H, NH-CH_2), 3.71 (m, 2H, N-CH_2), 5.63 (dd, ^{2}J = 1.36 Hz, ^{3}J = 10.34 Hz, 1H, =CH_2), 6.07 (dd, ^{3}J = 10.54 Hz, ^{3}J = 17.03 Hz, 1H, =CH), 6,13 (br, 1H, NH), 6.23 (dd, ^{2}J = 1.34 Hz, ^{3}J = 17.04 Hz, 1H, =CH_2). ^{13}C NMR (125 MHz, $CDCl_3$): δ (ppm)= 8,7 (CH_3), 37,4 (HN-CH_2), 39,7 (N-CH_2), 126,3 (=CH_2), 130,8 (=CH), 137,5 (C=C), 165,8 (HN-C=O), 172,5 (N-C=O).

Synthesis of adhesion promoter:

Step 1: 1-allyl-dimethyl-maleimide

5.0 g (39.6 mmol) 2,3-Dimethylmaleic anhydride and 11.3 g (198 mmol) allylamine were dissolved in 50 ml toluene. The mixture was heated at 130 °C for 3.5 h under and refluxed with a water trap. The solvent and the excess allylamine were evaporated under vacuum. The crude product was purified by column chromatography with silica gel and ethyl acetate/*n*-hexane 1:2 to yield 6.26 g of oil Yield: 96%. ^{1}H NMR ($CDCl_3$, 500 MHz): δ (ppm) = 1.95 (s, 6H, CH_3), 4.07 (d, J = 5.5 Hz, 2H, N-CH_2), 5.13 (m, 2H, CH_2), 5.77 (m, 1H, CH). ^{13}C NMR ($CDCl_3$, 126 MHz): δ (ppm) = 8.6 (CH_3), 40.0 (N-CH_2), 117.2 (CH_2), 132.0 (CH), 137.2 (C=C),171.7 (C=O).

Step 2: 1-[3-(chloro-dimethyl-silanyl)-propyl]-3,4-dimethylmaleimide

In a heated flask 0.31 g (1.8 mmol) of 1-allyl-dimethyl-maleimide and three drops of a 3 wt% solution of platinium (0)-1,3-divinyl-1,1,3,3-tetramethyldisiloxane-complex in xylene were added in 2 ml (18 mmol) monochlorodimethyl silane under argon atmosphere at 0 °C. The mixture was stirred for 1 h at 0 °C and subsequently overnight at room temperature. The solution was evaporated in vacuum to remove the excess monochlorodimethyl silane and give in quantitative yield. ^{1}H NMR ($CDCl_3$, 500 MHz): δ (ppm) = 0.39 (s, 6H, Si-CH_3), 0.78 (m, 2H, Si-CH_2), 1.67 (m, 2H, CH_2), 1.95 (s, 6H, CH_3), 3.48 (t, J = 4.2 Hz, 2H, N-CH_2). ^{13}C NMR ($CDCl_3$, 126 MHz): δ (ppm) = 1.5 (Si-CH_3), 8.6 (CH_3), 16.1 (Si-CH_2), 22.5 (CH_2), 40.3 (N-CH_2), 137.1 (C=C), 172.2 (C=O).

Table S1. EDX analysis (atom-%) of the metal oxide layers shown in Figure 2.

metal oxide	thickness (μm)	C	O	Al	Mg
Al_2O_3	1.77	0.37	62.3	37.3	(-)
MgO	0.646	2.1	55.6	(-)	42.4

Table S2. Amounts of polymer and metal salts used for the preparation of Al_2O_3 and MgO by pre-mixing the polymer and salt.

polymer / mg	$Al(NO_3)_3 \cdot 9H_2O$	$Mg(NO_3)_2 \cdot 6H_2O$
100	200	
100	300	
100	400	
200	400	
200	600	
200	800	
150		300
150		450
150		600

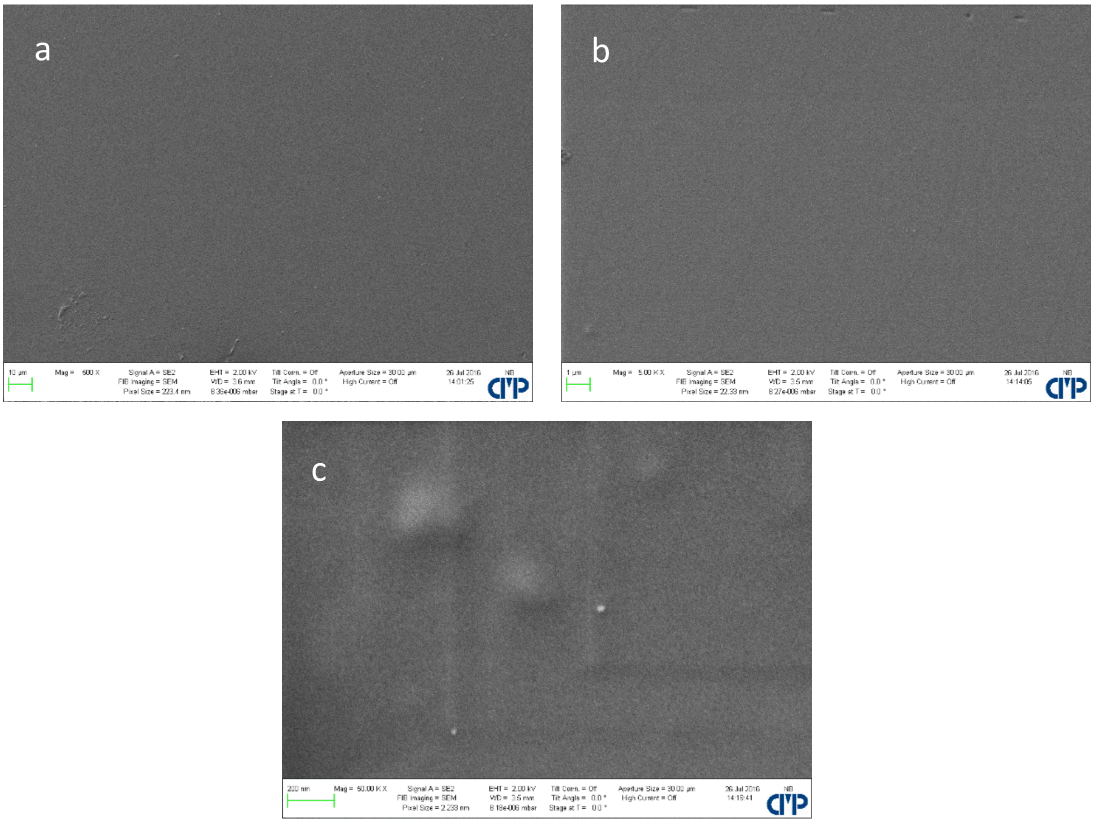

Figure S1. SEM images of dry hydrogel films prepared by spin-coating with variable polymer concentration and spin velocity (average film thicknesses: (a) 0.306 μm, (b) 0.607 μm, (c) 0.801 μm; see Table 1).

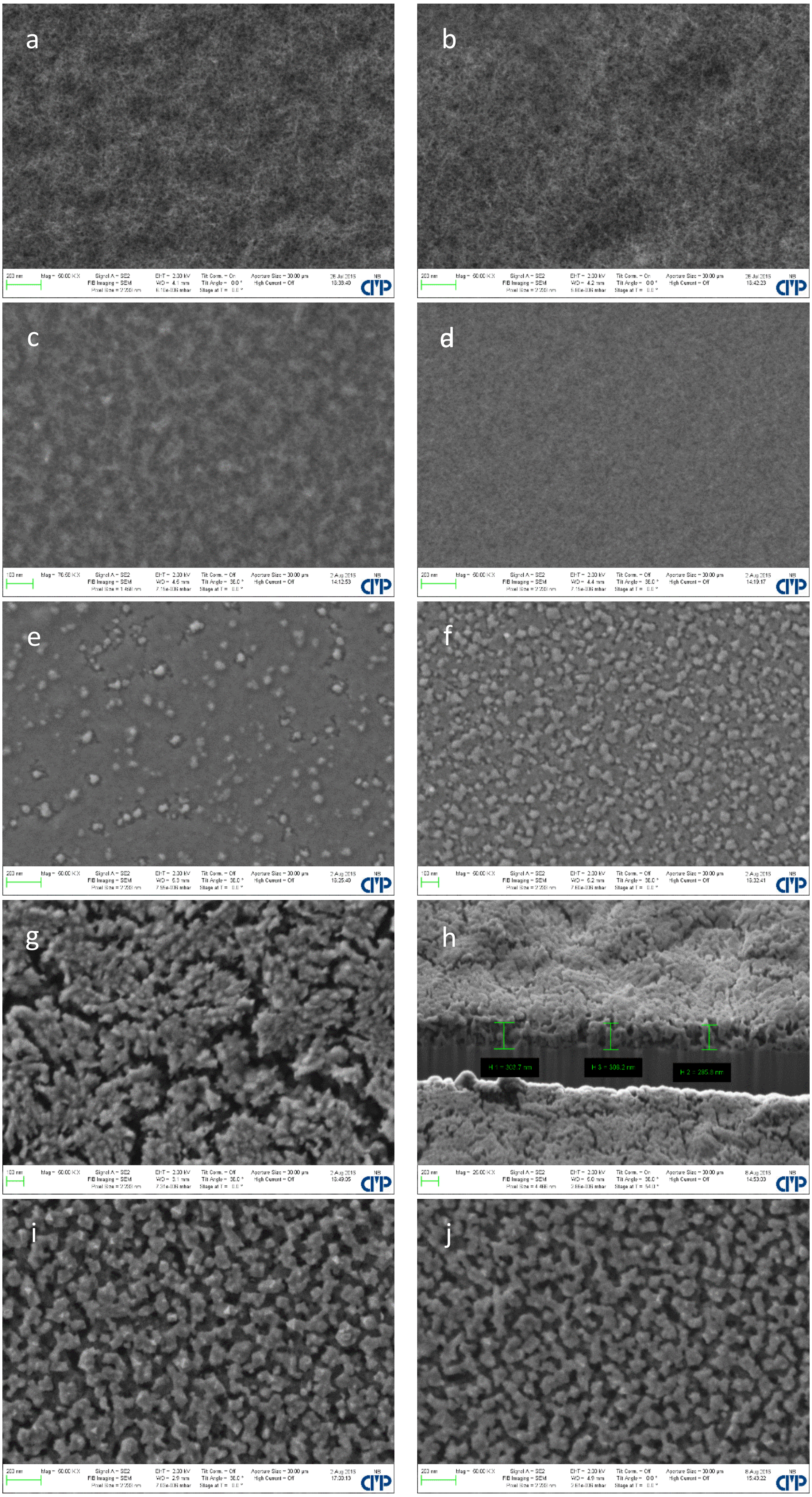

Figure S2. SEM images of (a-e) Al_2O_3 and (f-j) MgO prepared with hydrogel films of average thickness (μm): (a) 0.187, (b) 0.306, (c) 0.607, (d) 0.801, (e) 0.851, (f) 0.187, (g) 0.306, (h) 0.588, (i) 0.801, (j) 0.851.

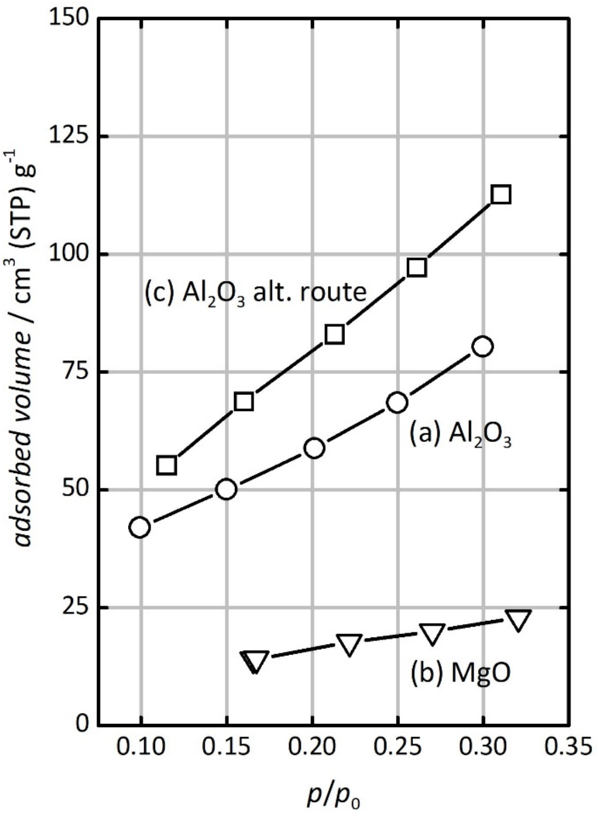

Figure S3. Kr physisorption isotherms of porous metal oxide layers: (a) Al_2O_3 layer (1.77 μm thickness), (b) MgO (0.646 μm), (c) Al_2O_3 (prepared by pre-mixing the polymer)

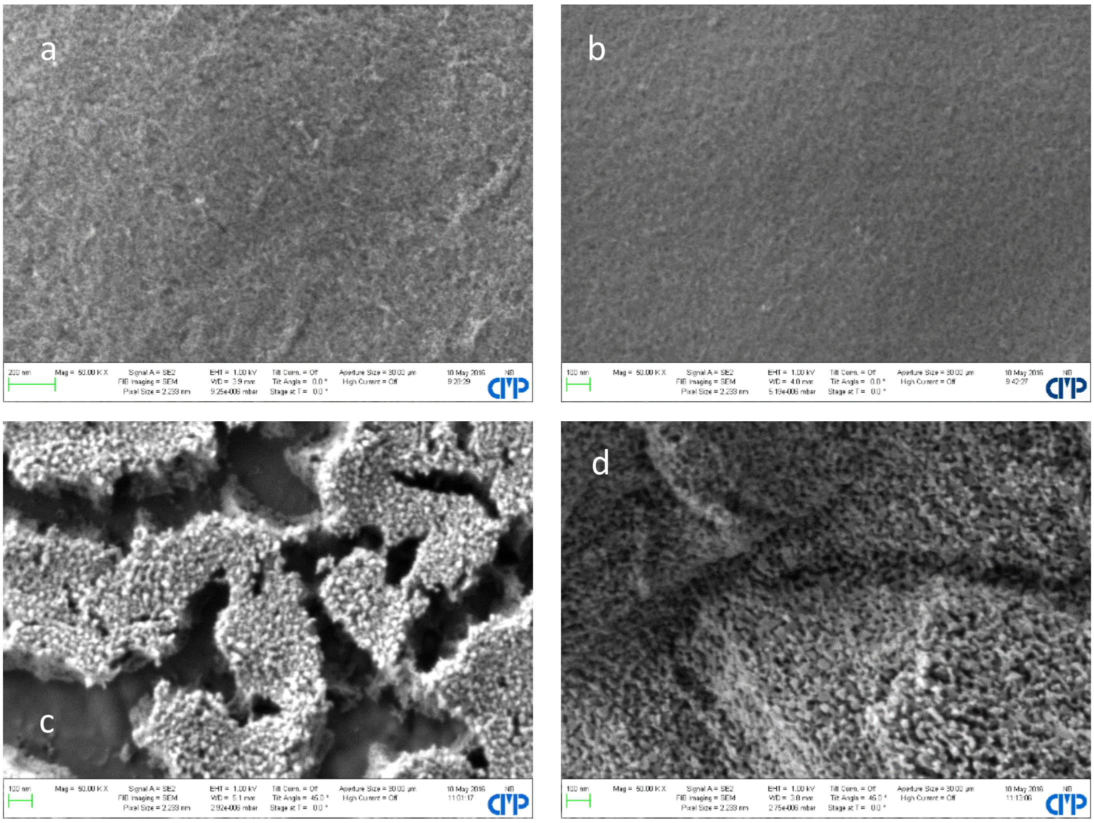

Figure S3. SEM images of (a,b) Al_2O_3 and (c,d) MgO prepared by pre-mixing the polymer (a,b: 100 mg) with the metal salts (a: 200 mg, b: 400 mg $Al(NO_3)_3 \cdot 9H_2O$; c: 300 mg, d: 600 mg $Mg(NO_3)_2 \cdot 6H_2O$) prior to spin-coating and subsequent photo-cross-linking and calcination.
